# Characterization of the complete chloroplast genome of the Taiwan alder *Alnus formosana* (Betulaceae) based on next-generation sequencing technology

**DOI:** 10.1080/23802359.2021.1969694

**Published:** 2021-09-06

**Authors:** Xiaoning Zhang, Mimi Li, Qiulan Wei, Yufei Xiao, Yufeng Qin, Lianxiang Zhong, Zihai Qin

**Affiliations:** a Guangxi Forestry Research Institute, Nanning, China; b Institute of Botany, Jiangsu Province and Chinese Academy of Sciences, Nanjing, China; c The Jiangsu Provincial Platform for Conservation and Utilization of Agricultural Germplasm, Nanjing, China

**Keywords:** *Alnus formosana*, Betulaceae, chloroplast genome, phylogeny

## Abstract

*Alnus formosana* (Betulaceae) is an important ecological and economic deciduous tree species widely distributed throughout subtropical regions of Taiwan province, China. At the present study, the complete chloroplast genome of *A. formosana* was assumbled using next-generation sequencing technology. The complete chloroplast sequence is 161,029 bp in length, which consisted of a large single copy (LSC, 89,720 bp) and a small single copy (SSC; 19,205 bp) separated a pair of inverted repeats (IRs; 26,052 bp). The overall guanine-cytosine (GC) content was 36.4%. A total of 131 genes were annotated, including 85 protein-coding genes, 37 tRNAs, eight rRNAs and one pseudogene (ψ*ycf*1). The phylogenetic analysis fully resolved *A. formosana* in a clade with *A. japonica*. The plastome of *A. formosana* will provide informative genomic resources for further phylogenetic application and genetic improvement.

*Alnus formosana* (Burkill) Makino 1912, classified in the birch family (Betulaceae), is an important ecological and economic deciduous tree species(Li and Skvortsov 1999). It has a wide natural distribution throughout the subtropical regions in the Taiwan province, China (Liao and Weng 2002). It is characterized by fast-growing, nitrogen-fixing and wide ranging adaptability to adverse environmental conditions, and is, therefore, widely used as pioneer tree of afforestation (Pan et al. 2008). This species is also a source of excellent quality wood for furniture and industrial production. However, the phylogenetic position of *A. formosana* within *Alnus* has not yet been sufficiently resolved (Ren et al. 2010). Here, we report the complete chloroplast (cp) genome sequence of *A. formosana* using next-generation sequencing, which will lay the foundation for further phylogenetic application and genetic resolution.

Total genomic DNA was extracted from the fresh leaves of *A. formosana* cultivated in Guangxi Forestry Research Institute, Guangxi Province, China (22°55′30″N, 108°21′E) by modified hexadecyl trimethyl ammonium bromide (CTAB) method (Doyle and Doyle 1987). The specimen was deposited at Guangxi Forestry Research Institute (contact: Zihai Qin, 75455621@qq.com) under voucher number: 20210303002. The isolated genomic DNA was subsequently sequenced on an Illumina Hiseq X-ten platform (San Diego, USA) at Novogene (Beijing, China). The raw Paired-end (PE) reads were *de novo* assembled into the chloroplast genome using the perl script NOVOPlasty 4.3.1 (Dierckxsens et al. 2017) with default settings. The assembled genome was annotated via GeSeq (Tillich et al. 2017) and adjusted manually in Geneious 11.1.5 (Kearse et al. 2012).

The chloroplast genome of *A. formosana* has a circular structure with a length of 161,029 bp, comprising four parts: a LSC (89,720 bp), a SSC (19,205 bp) and a pair of IRs (26,052 bp). The overall guanine-cytosine (GC) content was 36.4%, with the GC contents of LSC, SSC and IRs at 34.1%, 30.1%, and 42.6%, respectively. The complete sequence data was submitted to National Center for Biotechnology Information (NCBI) (accession number MW865380). A total of 131 genes were annotated, including 85 protein-coding, 37 transfer RNA (tRNA), eight ribosomal RNA (rRNA) and one pseudogene (ψ*ycf*1). Among them, four rRNA (*4.5S*, *5S*, *16S* and *23S* rRNA), seven tRNA (*trnI-GAU*, *trnA-UGC*, *trnL-CAA*, *trnI-CAT*, *trnR-ACG*, *trnV-GAC*, *trnN-GTT*), and seven protein-coding genes (*rpl*2, *rpl*23, *ycf*2, *ndh*B, *rps*7, *rps*12, and *ycf*15) were duplicated in the IR regions. The plastome of *A. formosana* had similar in the terms of structure, gene content and order, and GC content compared to those of other published *Alnus*.

To reveal the phylogenetic relationship of *A. formosana* within *Alnus*, we downloaded additional plastomes from NCBI including fifteen *Alnus* and one *Betula* species. The whole plastome sequences were aligned by MAFFT 7.409 (Katoh and Standley 2013) using default settings. The phylogenetic analysis was generated by the GTR + GAMMA model in RAxML with 1,000 bootstrap replicates (Stamatakis 2014). The maximum likelihood (ML) phylogenetic tree indicated that *Alnus* is a monophyletic group which is consistent with the previous analysis by Chen and Li (2004) but with higher bootstrap support (100%) (Figure 1). Within genus *Alnus*, *A. formosana* is most closely related to *A. japonica* (NC_036753) with a 100% bootstrap value, which is in accordance with their morphological features (Ren et al. 2010).

**Figure 1.**
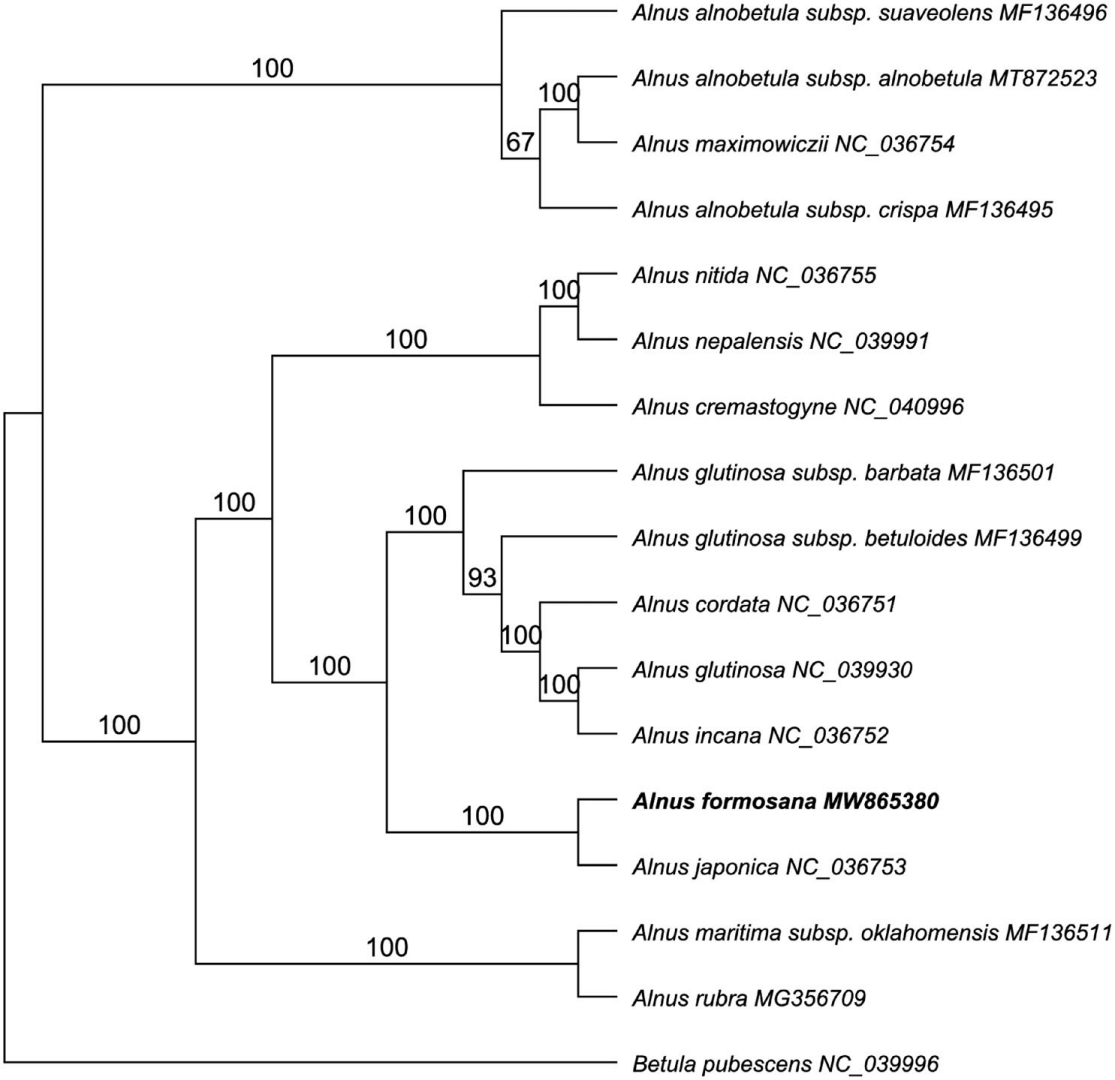
Phylogenetic tree illustrating the relationship of *Alnus formosana* using maximum likelihood with 1000 bootstrap replicates.

## Data Availability

The genome sequence data that support the findings of this study are openly available in GenBank of NCBI at (https://www.ncbi.nlm.nih.gov/) under the accession no. MW865380. The associated BioProject, SRA, and Bio-Sample numbers are PRJNA743224, SRR15021752 and SAMN20003570, respectively.
